# Editorial: Association of physical activity and fitness with mental health outcomes: Current advances and future directions

**DOI:** 10.3389/fpubh.2022.1027395

**Published:** 2022-09-15

**Authors:** Riki Tesler, Andreas Ihle, Adilson Marques

**Affiliations:** ^1^Department of Health Systems Management, School of Health Sciences, Ariel University, Ariel, Israel; ^2^Department of Psychology, University of Geneva, Geneva, Switzerland; ^3^CIPER, Faculdade de Motricidade Humana, Universidade de Lisboa, Lisbon, Portugal

**Keywords:** physical activity, fitness, mental health, disability, gender

Mental health disorders prevalence has been rising among children, adults, and older adults for the last two decades (1). These disorders are the leading causes of disability worldwide (2). Despite mental health disorders affect people of different ages and sex, it is almost twice as common in women than in men (1). The difference in the prevalence between men and women has been linked to biological and psychological sex differences in susceptibility and environmental factors operating on micro and macro levels (3). For instance, women undergo internal endocrine processes associated with the reproductive cycle, increasing the risk of depression during reproductive age (4). Furthermore, mental health disorders increase vulnerability and mortality, reducing life expectancy (5), mainly due to cardiovascular disease risk (6). Thus, an increased economic burden is expected due to treatment costs and productivity loss (7). Therefore, finding new strategies to prevent mental health disorders must be a priority of public health authorities.

Physical activity and fitness are consistently associated with reduced mental health disorders (8–10). Evidence suggests that physical activity and healthy fitness levels increase the quality of life and wellbeing (11–15). Engaging in physical activity is associated with benefits related to physical and mental health (16, 17). In addition to mitigating the functional decline and risk of disability (17, 18), physical activity causes changes in brain neurotransmitters and endogenous opioids associated with depression, anxiety and other mental health problems, enhancing the relationship between physical activity and quality of life (11).

People with high physical activity and healthy fitness levels have a lower risk of mental health disorders compared to those with lower physical activity and fitness levels (8). Consequently, the World Health Organization's physical activity guidelines recommend regular physical activity, mainly aerobic activities, to prevent several chronic diseases and mental health disorders (19). However, the adult population's PA (physical activity) levels are low (20).

Considering the effect of physical activity and physical fitness on mental health outcomes, the known state of the art at present and the directions for the future are important. Therefore, we carried out this special issue, with important contributions from several research groups around the world on this challenging topic.

Physical activity can be interpreted on different levels, and so Kim and Koo studied how the level of exercise affected mental health and quality of life (QOL) of depressed men related to their PA levels. What they found was that not all types of physical activity improved QOL. Flexibility, for instance, did not show significant improvements, which can be due to the fact that in South Korea it is associated with a feminine type of sports and even considered to be boring. Overall, the study shows that walking and strength exercise positively affected health-related QOL. In another study by Santos et al. depression symptoms were more severe when European adults spend more time watching TV. These higher levels of depressive symptoms could be decreased with increasing PA. This was the case for both men and women. A similar result was observed by Du et al. in individuals with sleep deprivation. When looking at the planning ability and alertness of these participants, the exercise gave better results on specific tasks than a nap of similar duration. This is interesting since students are a group known to have sleep deprivation. Ahmadabadi looked at this specific group during the COVID-19 pandemic and compared their anxiety and general-social health levels in students with more or less PA. She observed that regular exercise and an active life improved student's general performance and even physical health.

Yan et al. looked at adults with intellectual disabilities and concluded that those are a group that lacks physical activity. It would be highly beneficial for them to improve muscle and cardiopulmonary fitness. So far, the discussed studies have put their focus on adults and PA, but there is another important group to discuss, and that is the youth. Bowling et al. did a thorough literature review on youth and in particular the youth with social, emotional, and behavioral disabilities (SEBD). They show that for this target group, there are a handful of reasons causing them to lack physical activity compared to youth without these disabilities. As seen before in adults with various backgrounds, youth with SEBD benefit from physical activity. Physical activity can improve their behavior and several different aspects. Bowling et al. developed the Pediatric Physical Activity Engagement for Invisible Social, Emotional, and Behavioral Disabilities framework. Within this framework, physical activity is encouraged and tackled from all directions, like overcoming cultural differences, making the children feel included and adapting the physical activity to the exact needs of the exercise every individual needs. It creates a whole new engagement framework supporting children to benefit from physical activity.

All in all, from all around the world, across gender, age and mental abilities physical activity has a positive effect on mental and physical health. In this special issue, we would like to emphasize this importance and stimulate further detailed research efforts to capture the underlying mechanisms in future investigations as well as encourage individuals, young and old, men or women, from all mental abilities and regardless of their background, to try to find the appropriate place and time to work toward an active lifestyle.

## Author contributions

All authors listed have made a substantial, direct, and intellectual contribution to the work and approved it for publication.

## Conflict of interest

The authors declare that the research was conducted in the absence of any commercial or financial relationships that could be construed as a potential conflict of interest.

## Publisher's note

All claims expressed in this article are solely those of the authors and do not necessarily represent those of their affiliated organizations, or those of the publisher, the editors and the reviewers. Any product that may be evaluated in this article, or claim that may be made by its manufacturer, is not guaranteed or endorsed by the publisher.
